# Specific Association Patterns Between Brain Glutathione Levels, Myelination, and Functional Connectivity in Adults With Autism Spectrum Disorder

**DOI:** 10.1002/aur.70134

**Published:** 2025-10-23

**Authors:** Toshiki Iwabuchi, Takaharu Hirai, Naoko Umeda, Hideto Yogo, Yuuta Nishimiya, Yuuki Nishigaki, Masaru Watanabe, Hidenori Yamasue, Masatsugu Tsujii, Kenji J. Tsuchiya, Hideo Matsuzaki

**Affiliations:** ^1^ Research Center for Child Mental Development Hamamatsu University School of Medicine Shizuoka Japan; ^2^ United Graduate School of Child Development The University of Osaka, Kanazawa University, Hamamatsu University School of Medicine, Chiba University and University of Fukui Osaka Japan; ^3^ Department of Psychiatric and Mental Health Nursing, School of Nursing University of Fukui Fukui Japan; ^4^ Life Science Innovation Center University of Fukui Fukui Japan; ^5^ Department of Maternal and Child Health Nursing, School of Nursing University of Fukui Fukui Japan; ^6^ Kojin Hospital Nagoya Aichi Japan; ^7^ Department of Psychiatry Hamamatsu University School of Medicine Shizuoka Japan; ^8^ School of Contemporary Sociology Chukyo University Nagoya Aichi Japan; ^9^ Research Center for Child Mental Development University of Fukui Fukui Japan

**Keywords:** autism spectrum disorder, glutathione, magnetic resonance spectroscopy, myelin map, resting‐state functional connectivity

## Abstract

Recent studies have implicated oxidative stress in the pathophysiology of autism spectrum disorder (ASD). Postmortem brain studies have revealed decreased levels of the reduced form of glutathione (GSH), an important antioxidant, in some brain regions in individuals with ASD; however, in vivo evidence is lacking. Using proton magnetic resonance spectroscopy, T_1_‐weighted/T_2_‐weighted ratio‐derived myelin maps, resting‐state functional magnetic resonance imaging (MRI), and cognitive tasks, we examined whether brain GSH levels are lower in individuals with ASD than in those with typical development (TD) and explored ASD‐specific association patterns between brain GSH levels, myelination, functional connectivity, and behavioral characteristics. Data from 30 adults with ASD and 27 adults with TD were analyzed. Contrary to our hypothesis, GSH levels in the left temporoparietal junction (TPJ) were higher in the ASD group than in the TD group. Using individual myelin maps, we found a significant group difference in the correlation between left middle frontal gyrus (MFG) myelination and left TPJ GSH levels. Multivariate pattern analysis of resting‐state functional MRI revealed that whole‐brain functional connectivity patterns from the left MFG differed between the groups in their association with left MFG myelination. Finally, we found a significant group difference in the correlation between emotion recognition ability and the functional connectivity of the left MFG with the bilateral occipitoparietal junction. In conclusion, our findings demonstrate an ASD‐specific pattern of associations between left TPJ GSH levels, left MFG myelination, whole‐brain functional connectivity patterns of the left MFG, and cognitive phenotype, which suggests compensatory neural mechanisms in ASD.

**Summary**

- It has been suggested that people with autism spectrum disorder (ASD) have lower levels of antioxidants—that protect cells from damage—in the brain.
- However, we found that individuals with ASD exhibited a higher concentration of one of these protective antioxidants, glutathione, in a specific brain area than their non‐ASD peers.
- This increase may be related to changes in brain networks that could affect how emotions are recognized. Our findings provide new insights into the biological mechanisms underlying ASD.

## 1 Introduction

Autism spectrum disorder (ASD), characterized by difficulties in social communication and repetitive and restricted patterns of behaviors (American Psychiatric Association 2013), is a neurodevelopmental disorder of which the underlying neurobiological mechanisms remain unknown. Epidemiological studies have demonstrated an increasing trend in ASD diagnoses; the Centers for Disease Control and Prevention revealed a 1 in 36 (2.8%) prevalence among 8‐year‐old children in 2020 (Maenner et al. 2023), which was higher than that in 2018 (1 in 44; 2.3%) (Maenner et al. 2021). Despite the increasing global burden of ASD, reliable pharmacological interventions are yet to be established. Revealing the neurobiological underpinnings of behavioral features of ASD and identifying effective therapeutic targets are urgently needed.

Mounting evidence has suggested that redox imbalance and the resulting increase in oxidative stress may be important factors in ASD (Usui et al. 2023). Particularly, the altered metabolism of glutathione, the most abundant endogenous antioxidant in the brain, may play a key role in ASD etiology through increased oxidative stress and/or mitochondrial dysfunction caused by a glutathione redox imbalance (Bjørklund et al. 2020). Meta‐analyses have demonstrated decreased levels of the reduced form of glutathione (GSH) in blood samples of individuals with ASD (Chen et al. 2021; Frustaci et al. 2012). Additionally, postmortem brain studies have revealed decreased GSH levels in the temporal cortex and cerebellum (Chauhan et al. 2012; Rose et al. 2012). Given that several clinical trials have suggested *N*‐acetylcysteine, a precursor of GSH, as a potential treatment for ASD symptoms (Deepmala et al. 2015), elucidating the relationship between atypical GSH metabolism and ASD symptomatology is critical for establishing new and effective interventions.

However, limited in vivo evidence exists for atypical GSH levels in individuals with ASD. To date, five proton magnetic resonance spectroscopy (^1^H‐MRS) studies have been published, with none revealing significant differences in GSH levels between individuals with ASD and those with typical development (TD) (Durieux et al. 2016; Endres et al. 2017; Pereira et al. 2024; Sapey‐Triomphe et al. 2021; Song et al. 2024). However, the regions of interest (ROIs) used in these studies, such as prefrontal cortices or basal ganglia, differed from those examined in postmortem brain studies (the temporal cortex and cerebellum). Given the differences between previous MRS and postmortem brain studies, the non‐significant MRS findings in previous studies may reflect the regional specificity of atypical GSH metabolism in ASD.

Consistent reports of functional or structural alterations in individuals with ASD within or around the temporal cortex, particularly in the temporoparietal junction (TPJ), suggest that this region may be responsible for social difficulties in individuals with ASD (Patriquin et al. 2016). Similarly, converging evidence suggests that the cerebellum also plays a significant role in the pathophysiology of ASD (Kelly et al. 2020). Thus, we expected that altered GSH levels can be detected in these regions. In addition, the pregenual anterior cingulate cortex (pgACC) is another candidate region for ASD‐related GSH alteration. Using positron emission tomography (PET), our previous study found decreased mitochondrial complex I activation in adults with ASD compared with TD controls (Kato et al. 2023), and mitochondrial dysfunction and oxidative stress are closely interrelated (Guo et al. 2013; Okoye et al. 2023). Moreover, functional neuroimaging and MRS studies have also demonstrated functional and neurometabolic differences in this region between individuals with ASD and those with TD (Bejjani et al. 2012; Di Martino et al. 2009). Since GSH levels in these regions have not been investigated in previous MRS studies focusing on ASD, the present study aimed to examine GSH levels in the TPJ, cerebellum, and pgACC.

Reduced GSH levels in the brain can cause myelin deformation, which reduces the effectiveness of information transfer in functional networks. Oligodendrocytes, which regulate myelination, are vulnerable to oxidative stress in rats (French et al. 2009; Thorburne and Juurlink 1996). The results of a previous study indicated that the anti‐adenomatous polyposis coli clone CC1, a marker of mature oligodendrocytes, was reduced in the anterior cingulate cortex of knockout mice in genes coding for modifier subunits of glutamate‐cysteine ligase as a consequence of GSH deficiency (Monin et al. 2015). The same study showed that GSH levels in the medial prefrontal cortex were associated with fractional anisotropy, which may partly reflect the degree of white matter myelination and functional connectivity along the human cingulum bundle in healthy individuals (Monin et al. 2015). Notably, this study showed that the correlation patterns between GSH levels and functional connectivity differed between patients with early psychosis and healthy controls. In the patient group, medial frontal GSH levels were associated with fractional anisotropy but not functional connectivity.

Comparing the ASD and TD groups, the present study focused on such differences in the correlation patterns between different neuroimaging measures. As our previous study suggested (Murayama et al. 2022), group differences in the correlation patterns between molecular features (e.g., dopamine D2/3 receptor availability) and other neuroimaging features (e.g., functional connectivity) may indicate neurobiological or compensatory mechanisms underlying the differences between these populations. In the brains of individuals with ASD, altered GSH levels in one region may affect myelination differently than in those with TD, possibly leading to different correlation patterns between GSH levels and myelin‐related measures in the brain.

Altered myelination can subsequently lead to changes in functional connectivity in the brain, given the systematic associations between these measures (Baum et al. 2020; Huntenburg et al. 2017). Previous studies using multimodal neuroimaging techniques (e.g., PET and functional magnetic resonance imaging [fMRI]) have shown that molecular alterations (e.g., altered dopamine D2/3 receptor availability) in a specific region can impact functional networks comprising distant regions (Nour et al. 2019; Murayama et al. 2022). Given these potential long‐distance associations between myelination and functional connectivity, we investigated group differences in the correlations between regional myelination and whole‐brain functional connectivity alterations. Ultimately, altered functional networks may underlie the behavioral differences between the ASD and TD groups.

The present study aimed to investigate a mechanistic pathway in which (1) brain GSH levels are reduced in individuals with ASD compared to their TD peers, leading to (2) changes in myelination, which in turn (3) affect functional connectivity in an ASD‐specific manner, and (4) contribute to the behavioral characteristics of ASD. We used a multimodal MRI approach with ^1^H‐MRS, T_1_‐weighted/T_2_‐weighted (T_1_w/T_2_w) ratio‐derived myelin map analysis, resting‐state functional MRI (rsfMRI), and cognitive tasks to examine whether (1) individuals with ASD have reduced GSH levels in regions functionally involved in ASD, (2) GSH levels in these regions are differentially associated with myelination between the ASD and TD groups, (3) the correlation patterns between myelination and functional connectivity differ between the groups, and (4) functional connectivity is differentially associated with behavioral phenotypes between the groups.

## 2 Methods

### 2.1 Participants

This study was approved by the Ethics Committees of the University of Fukui (Protocol No. 20200112) and Hamamatsu University School of Medicine (Protocol No. 20‐258) and conducted in accordance with the principles of the Declaration of Helsinki. Written informed consent was obtained from all the participants.

Thirty‐two adults with a diagnosis of ASD and 44 with no history of neurodevelopmental or psychiatric disorders were recruited. The target sample size for the analysis was originally set at 30 for each group, based on previous studies (Durieux et al. 2016; Endres et al. 2017) and the practical feasibility of recruitment. All the participants underwent psychological assessments, including assessments of autistic traits using the Social Responsiveness Scale, Second Edition (SRS‐2) (Constantino and Gruber 2012) and full‐scale intelligence quotient (IQ) using the Wechsler Adult Intelligence Scale—Fourth Edition (WAIS‐IV) or Third Edition (WAIS‐III) (Wechsler 1997, 2008). For participants in the ASD group, the Autism Diagnostic Observation Schedule, Second Edition (ADOS‐2) was administered by a researcher (TH) with an ADOS‐2 research license. The data of participants in the ASD group were excluded from the analysis if their ASD diagnosis was not confirmed using the ADOS‐2 assessment. Regarding the TD group, the data of those with an SRS‐2 total score ≥ 55 (Kamio et al. 2013) were excluded from the analysis owing to high autistic traits. Although the TD group did not reach the target sample size, recruitment had to stop because the MRI scanner used in this study was no longer available. The socioeconomic status (SES) of the participants and their parents was assessed using the Hollingshead scale (Hollingshead 1957). Statistical comparisons between groups were conducted on background information using a two‐sample *t*‐test for continuous variables and a chi‐square test for categorical variables. Other background information is provided in the Supplementary Methods.

### 2.2 MRI and MRS Data Acquisition

A 3 T‐MR scanner (Signa HDxt; GE Healthcare, Milwaukee, WI) with an 8HRBRAIN coil at Kojin Hospital (Nagoya, Japan) was used to obtain T_1_‐ and T_2_‐weighted structural images, rsfMRI, and ^1^H‐MRS. T_1_‐weighted images were collected as references for spatial normalization. We also acquired T_2_‐weighted images to create individual myelin maps by calculating the T_1_w/T_2_w ratio (Glasser and Van Essen 2011). T_2_*‐weighted gradient‐echo echo‐planar images were acquired as rsfMRI data (Supplementary Methods).

The Supplementary Methods detail the ^1^H‐MRS acquisition parameters. Based on previous postmortem brain studies, GSH levels were assessed in various regions, including the temporal cortex and cerebellum. For the temporal cortex ROI, we focused on the TPJ, including the posterior end of the temporal cortex and the inferior parietal lobule. In addition to the bilateral TPJ and cerebellum, we defined the pgACC ROI. Detailed information and examples related to ROIs are provided in the Supplementary Methods and Figure S1a.

### 2.3 Cognitive Phenotypic Data Acquisition and Analysis

To examine potential associations between atypical functional networks and cognitive phenotypes, the participants underwent three cognitive tasks (Supplementary Methods). We used the Reading the Mind in the Eyes Test (RMET) as an index of emotion recognition ability, which may be a cognitive phenotype associated with social difficulties in individuals with ASD (Baron‐Cohen et al. 2001, 2015). The Japanese version of the RMET (Sato et al. 2016, 2017) was performed, and the accuracy was obtained. As proxies for perceptual inflexibility, which may be associated with repetitive and restricted behavioral patterns in ASD, we conducted the binocular rivalry task (Robertson et al. 2013, 2016) and the Necker lattice task (Kornmeier et al. 2017). The perceptual switching rate was obtained for each task. For statistical comparisons between groups, cognitive phenotypic data were subjected to two‐sample *t*‐tests.

### 2.4 MRS Data Analysis

Using LCModel (Version 6.3‐1K; http://s‐provencher.com/lcmodel.shtml) (Provencher 1993, 2001), water‐referenced quantification of GSH levels in the pgACC, right and left TPJ, and cerebellum was estimated (Supplementary Methods). Figure S1b shows a sample spectrum.

### 2.5 Myelin Map Analysis

A myelin map was created by calculating the ratio of T_1_‐ and T_2_‐weighted images on a voxel‐by‐voxel basis (Glasser and Van Essen 2011). For the myelin map analysis, we used MRTool (v.1.4.3; https://www.nitrc.org/projects/mrtool/), implemented in the SPM12 (https://www.fil.ion.ucl.ac.uk/spm/software/spm12/) (Supplementary Methods).

Statistical analysis was conducted using the general linear model (GLM) framework. SPM12 was used to create a design matrix and estimate the coefficients. The analysis was aimed at exploring the regions showing ASD‐specific association patterns between atypical GSH levels and myelination by examining the interaction between GSH levels and groups (with or without an ASD diagnosis). Therefore, we used the myelin content indexed by the T_1_w/T_2_w ratio in each voxel as a dependent variable. Categorical variables for the two groups were included as independent variables. GSH levels were extracted from the regions showing significant group differences, and the interaction term between the groups and GSH levels was also included in the GLM analysis. We explored regions showing significant interaction using the voxel‐level threshold of *p* < 0.001 (uncorrected for multiple comparisons, one‐tailed) and the cluster‐level threshold of *p* < 0.05 (false discovery rate–corrected, one‐tailed).

### 2.6 rsfMRI Data Preprocessing

The CONN toolbox version 20.b (Whitfield‐Gabrieli and Nieto‐Castanon 2012) and SPM12 were used to analyze the rsfMRI data. Preprocessing and data denoising were conducted using a standard pipeline implemented in the CONN toolbox (Supplementary Methods).

### 2.7 Multivariate Pattern Analysis: Associations Between T_1_w/T_2_w Ratio and Resting‐State Functional Connectivity

We adopted MVPA (Nieto‐Castanon 2022) (implemented in the CONN toolbox) to examine group differences in the associations between myelination (T_1_w/T_2_w ratio) and functional connectivity (Supplementary Methods). MVPA is a data‐driven method developed to identify seed regions for seed‐to‐voxel functional connectivity analyses. The multivariate functional connectivity patterns from each voxel to the rest of the brain were computed using the MVPA pipeline in the CONN toolbox. This produced individual functional connectivity maps for each voxel. Singular value decomposition was then used to reduce the dimensionality and create MVPA maps characterizing whole‐brain functional connectivity patterns from each voxel. Using MVPA‐derived maps as dependent variables, we conducted a multivariate analysis of covariance to examine the interaction effect between myelination (indexed as the T_1_w/T_2_w ratio) and the groups on whole‐brain functional connectivity patterns. Using significant clusters identified by MVPA as seeds, we performed a post hoc seed‐to‐voxel analysis to further illustrate the interaction.

### 2.8 Associations Between Resting‐State Functional Connectivity and Cognitive Phenotypes

Using the regions identified in the former MVPA as seed regions, a seed‐to‐voxel analysis was performed to investigate the associations between functional connectivity and cognitive phenotypes that differed between the groups. Along with the two group variables, interaction terms between the cognitive phenotypes and groups were included in the model, similar to the MVPA. Only cognitive phenotype scores that showed differences between the groups were used in this analysis. The statistical maps were thresholded at voxel and cluster levels of *p* < 0.001 (uncorrected, two‐tailed) and *p* < 0.05 (family‐wise error–corrected), respectively.

### 2.9 Sensitivity Analysis

As described later, a significant group difference was found in parental SES, but this was not adjusted for in the above statistical analyses because of missing data. Given the relatively limited sample size, we did not include parental SES as a covariate to avoid sample size reduction. As a sensitivity analysis, we confirmed the robustness of our findings when parental SES was statistically adjusted for.

### 2.10 Plasma Sample Analysis

Blood samples were collected to estimate plasma total glutathione levels (Supplementary Methods), which were compared between the groups. To examine the associations between GSH levels in the brain and the total glutathione levels in the blood samples, we calculated Spearman correlation coefficients between brain GSH levels assessed by ^1^H‐MRS and total glutathione levels in the plasma samples.

## 3 Results

### 3.1 Participant Demographics

Based on the exclusion criteria, 30 adults with ASD and 27 with TD were included (Supplementary Results). Table 1 and Table S1 present their demographic characteristics. Individuals with ASD showed significantly higher parental SES scores than did those with TD (*t* = 2.87, *p* = 0.006). However, age, biological sex, and full‐scale IQ scores were matched between the groups.

**TABLE 1:** Participants' background information.

Characteristics	ASD (*N* = 30)	TD (*N* = 27)	Statistics
	Mean (SD)	Mean (SD)	
Age (years)	29.7 (7.1)	29.0 (5.7)	*t* = 0.39, *p* = 0.70
Sex (male)	28	25	*χ* ^2^ = 0.012, *p* = 0.91
SES (self)	3.3 (0.9)^a^	2.3 (0.8)	*t* = 4.65, *p* < 0.001
SES (parent)	2.2 (0.5)^b^	2.7 (0.7)^c^	*t* = 2.87. *p* = 0.006
Full‐scale IQ	97.1 (24.0)	100.1 (9.4)	*t* = 0.60. *p* = 0.55
SRS‐2 total score	85.7 (32.2)	35.2 (13.0)	*t* = 7.59, *p* < 0.001
ADOS‐2			
Reciprocity	6.6 (2.0)		
Communication	11.9 (5.0)		
RRB	0.6 (0.9)		

Abbreviations: ADOS‐2, autism diagnostic observation schedule (second edition); ASD, autism spectrum disorder; IQ, intelligence quotient; RRB, repetitive/restricted behaviors; SD, standard deviation; SES, socioeconomic status; SRS‐2, social responsiveness scale (second edition); TD, typical development.

^a^ Data from one participant is missing.

^b^ Data from four participants is missing.

^c^ Data from one participant is missing.

### 3.2 Results of Cognitive Phenotypic Data Analysis

The RMET accuracy was significantly lower in the ASD group than in the TD group (*t* = 5.43, df = 51, *p* < 0.001, Cohen's *d* = 1.48). Other tasks related to perceptual rigidity showed no significant differences between the groups (Supplementary Results).

### 3.3 MRS Results: Group Differences in GSH Levels

Based on the criteria shown in the Supplementary Methods, 14 and 2 participants were excluded from the analyses for the pgACC ROI and right TPJ ROI, respectively. The pgACC ROI was excluded from the analysis because of unreliable data quality. The means ± SDs of full‐width at half‐maximum (FWHM) and signal‐to‐noise ratio (*S*/*N*) were as follows: left TPJ, FWHM = 0.045 ± 0.009, S/*N* = 37.51 ± 4.40; right TPJ, FWHM = 0.046 ± 0.008, S/*N* = 32.89 ± 9.57; cerebellum, FWHM = 0.062 ± 0.014, S/*N* = 28.56 ± 5.68.

**FIGURE 1:**
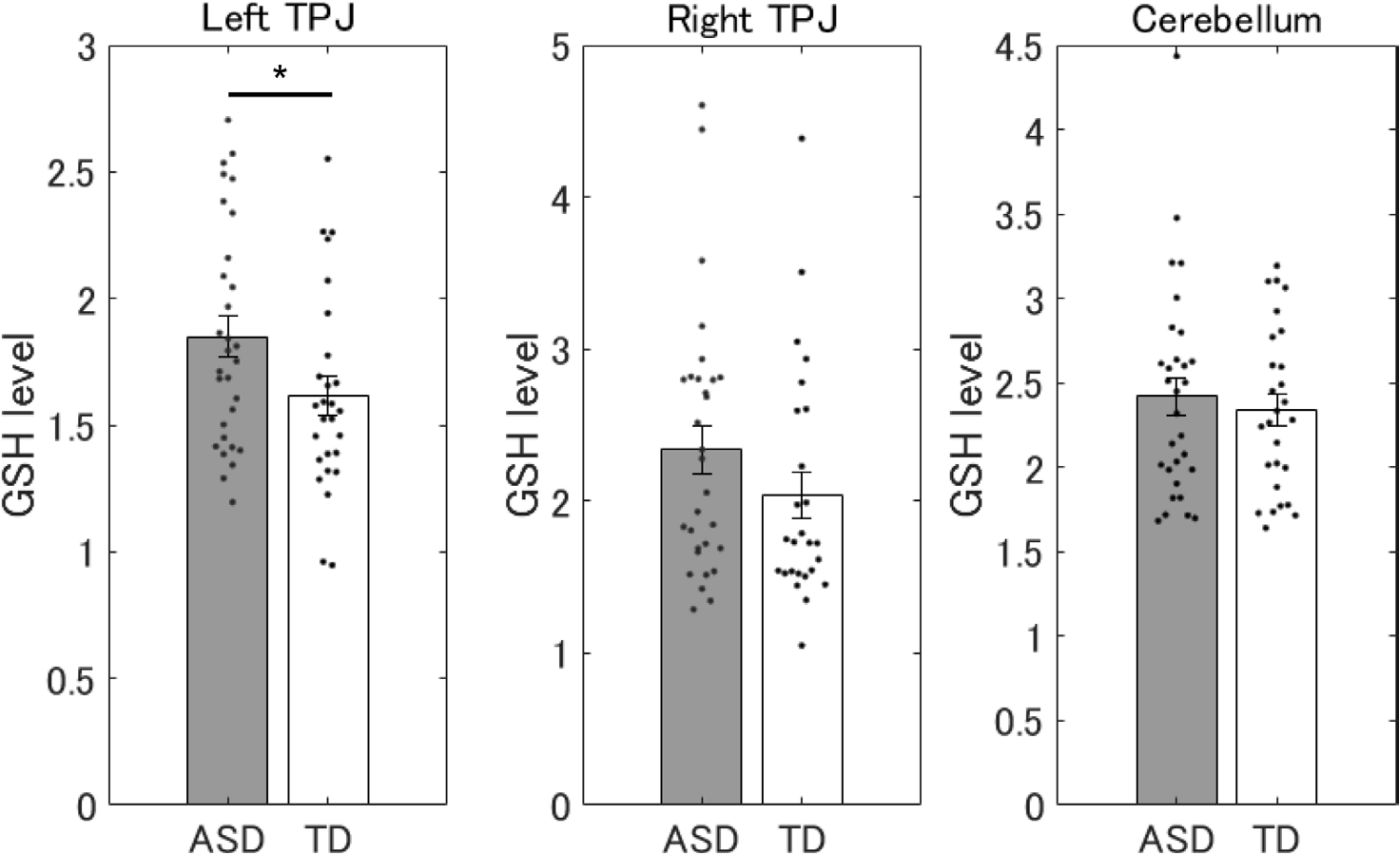
Magnetic resonance spectroscopy results. ASD, autism spectrum disorder; TD, typical development; TPJ, temporoparietal junction. *p < 0.05.

The GSH levels in the left TPJ were significantly higher in the ASD group than in the TD group (*t* = 2.12, df = 55, *p* = 0.038, Cohen's *d* = 0.56). No significant group differences were found in the cerebellum (*t* = 0.565, df = 55, *p* = 0.57, Cohen's *d* = 0.15) and right TPJ (*t* = 1.38, df = 55, *p* = 0.17, Cohen's *d* = 0.37) (Figure 1).

### 3.4 Myelin Map Results: ASD‐Specific Associations Between GSH Levels and T_1_w/T_2_w Ratio

**FIGURE 2:**
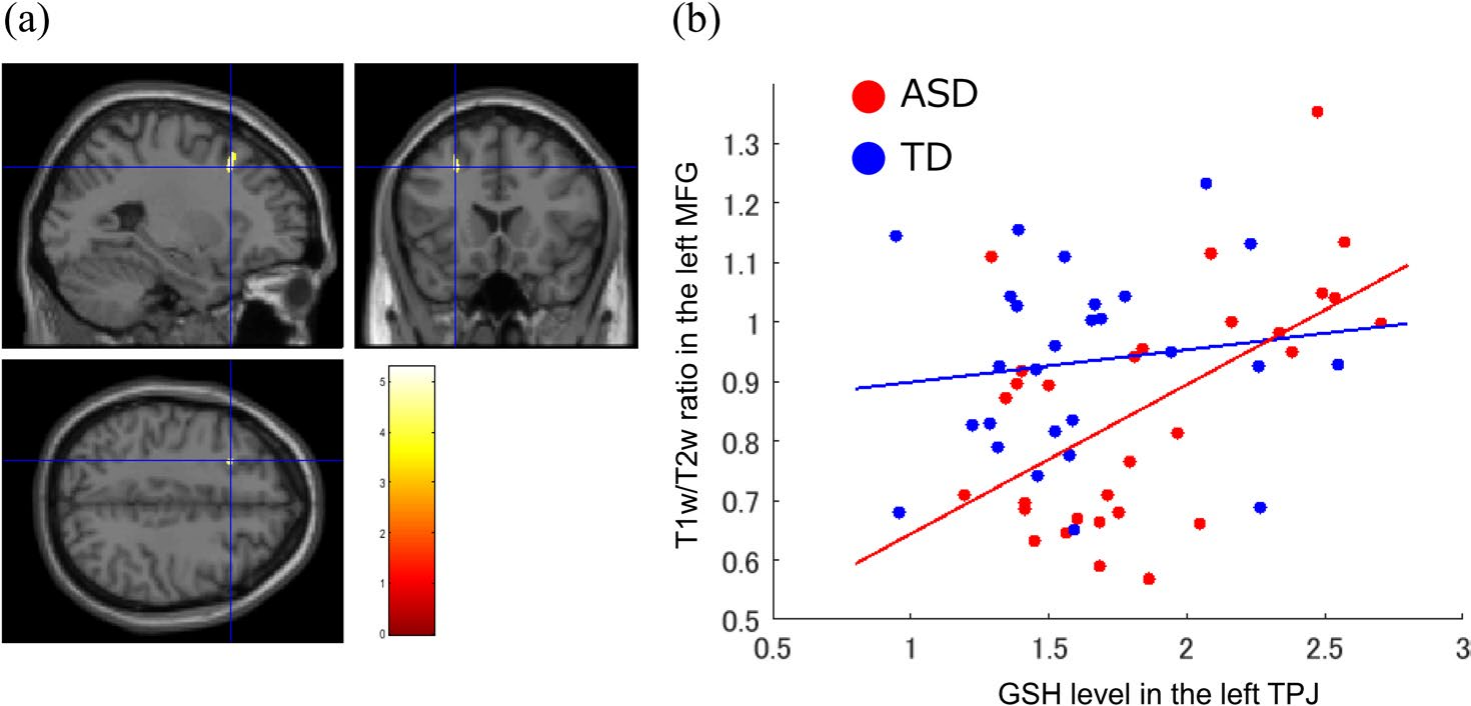
Myelin map results. (a) The left MFG is identified by the general linear model analysis (peak coordinate = [−26, 19, 42], cluster size = 130, *t* = 5.49). (b) Specifically, in the ASD group, the T_1_w/T_2_w ratio in the left MFG is positively correlated with left TPJ GSH levels. ASD, autism spectrum disorder; GSH, reduced form of glutathione; MFG, middle frontal gyrus; TD, typical development; TPJ, temporoparietal junction.

Only the GSH levels in the left TPJ were used in the myelin map analysis because no significant group differences were found in other regions. Here, we explored the regions where the associations between T_1_w/T_2_w ratios and left TPJ GSH levels differed between the groups. This interaction effect was identified in the left middle frontal gyrus (MFG) (peak coordinate = [−26, 19, 42], cluster size = 130, *t* = 5.49) (Figure 2a), where myelin content was positively correlated with left TPJ GSH levels specifically in the ASD group (Figure 2b).

### 3.5 MVPA Results: ASD‐Specific Associations Between T_1_w/T_2_w Ratio and Resting‐State Functional Connectivity

**FIGURE 3:**
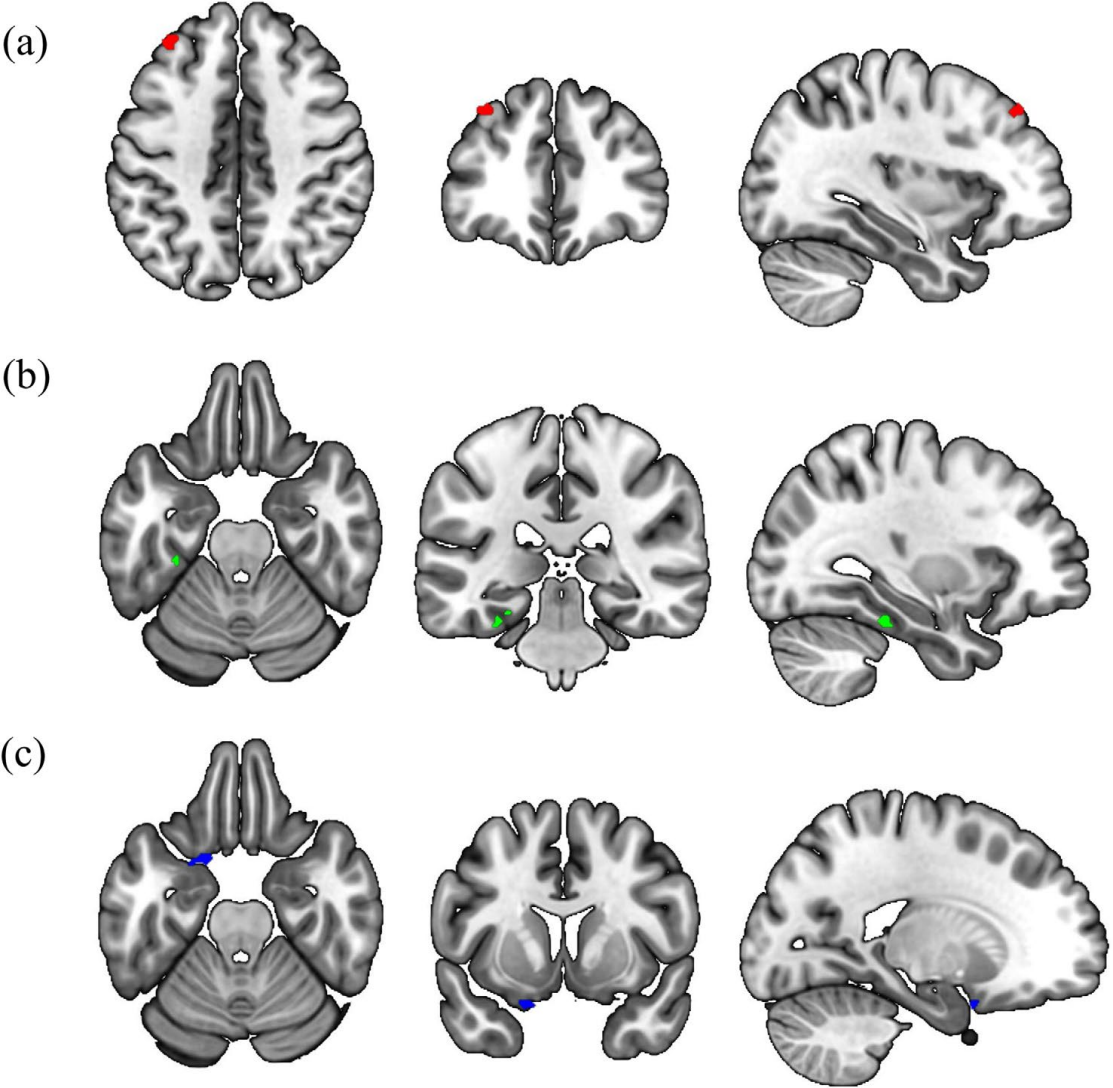
MVPA results. The (a) left MFG, (b) left fusiform gyrus, and (c) orbitofrontal cortex were identified by MVPA on rsfMRI data. MVPA, multivariate pattern analysis; MFG, middle frontal gyrus; rsfMRI, resting‐state functional magnetic resonance imaging.

The individual myelination values (T_1_w/T_2_w ratios) in the left MFG cluster, identified by the myelin map analysis, were used in the MVPA. The left MFG (peak coordinate = [−32, 38, 46], cluster size = 24), left fusiform gyrus (peak coordinate = [−30, −32, −24], cluster size = 22), and left orbitofrontal cortex (peak coordinate = [−18, 6, −24], cluster size = 20) showed significant interactions between the group and T_1_w/T_2_w ratio in the left MFG (Figure 3). Sensitivity analysis confirmed that the left MFG and left fusiform gyrus were identified even after adjusting for parental SES; thus, only these two regions were subjected to post hoc seed‐to‐voxel analysis.

Using the left MFG and left fusiform gyrus as seed regions, a post hoc seed‐to‐voxel analysis was performed to illustrate specific connectivity patterns showing the interaction between left TPJ GSH levels and ASD diagnosis. When the left MFG cluster was used as a seed region, resting‐state functional connectivity (rsFC) to the left middle temporal cortex showed a more positive correlation with left MFG myelination in the ASD group than in the TD group (Table S2 and Figure S2). Conversely, rsFC from the left MFG to the anterior cingulate cortex/dorsal medial prefrontal cortex, right putamen, bilateral supramarginal gyrus, right inferior frontal gyrus, and left occipital cortex, including the precuneus, showed a greater correlation with left MFG myelination in the ASD group than in the TD group (Table S2 and Figure S2). For the left fusiform gyrus seed, the precuneus/posterior cingulate cortex, rostral part of the medial prefrontal cortex, bilateral TPJ, bilateral MFG, and cerebellum were identified. The functional connectivity between these regions and the left fusiform gyrus was more negatively correlated with left MFG myelination in the ASD group than in the TD group (Table S2 and Figure S3).

### 3.6 Group Differences in Association Patterns Between rsFC and Cognitive Phenotypes

**FIGURE 4:**
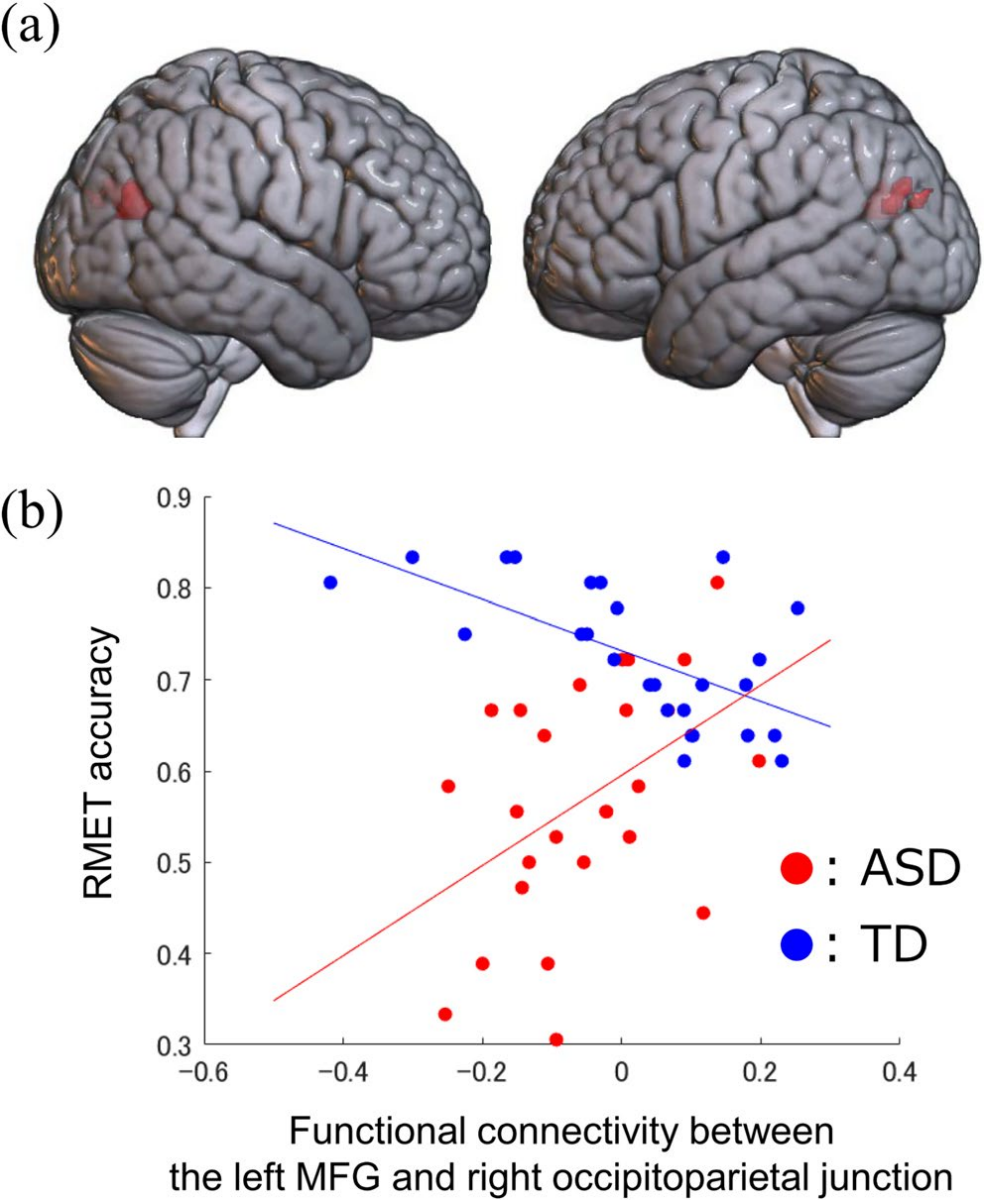
Group difference in the association between functional connectivity and emotion recognition ability. (a) Functional connectivity between the bilateral occipitoparietal junction and the left MFG is differentially correlated with the RMET accuracy between groups (right occipitoparietal junction, peak coordinate = [38, −68, 20], cluster size = 129; left occipitoparietal junction, peak coordinate = [−38, −70, 20], cluster size = 108). (b) Increased functional connectivity between these regions is positively correlated with emotion recognition ability in the ASD group, while this correlation is reversed in the TD group. ASD, autism spectrum disorder; MFG, middle frontal gyrus; RMET, the Reading the Mind in the Eyes Test; TD, typical development.

Only RMET data were used in this seed‐to‐voxel analysis because we found no significant differences between the groups in the other two tasks (the binocular rivalry and Necker lattice tasks). Using the left MFG cluster as a seed, we found that the rsFC with the bilateral occipitoparietal junction was differently correlated with the RMET score between the groups (Figure 4a). The rsFC from the left MFG to the right and left occipitoparietal junctions was positively correlated with the RMET score in the ASD group, whereas the correlation was negative in the TD group (right occipitoparietal junction, peak coordinate = [38, −68, 20], cluster size = 129; left occipitoparietal junction, peak coordinate = [−38, −70, 20], cluster size = 108) (Figure 4b). When the left fusiform gyrus was used as a seed, no significant interaction was identified.

### 3.7 Sensitivity Analysis Results

Sensitivity analyses confirmed the robustness of the results of the MRS analysis, myelin map analysis, MVPA (except for the left orbitofrontal cluster), and correlations between the left MFG rsFC and emotion recognition ability, even after adjustment for parental SES (Supplementary Results).

### 3.8 Results of Plasma Sample Analysis

We calculated Spearman correlations between total glutathione levels in the plasma sample and brain GSH levels in three ROIs (the cerebellum and bilateral TPJ), but no significant correlation was found (Supplementary Results and Table S3). To investigate the peripheral role of glutathione in ASD, we compared plasma total glutathione levels between the groups, but no significant difference was observed (*p* = 0.157; Figure S4). For reference, we also investigated the Spearman correlation between plasma total glutathione levels and other ASD traits and observed no significant correlations (Table S3).

## 4 Discussion

Using multimodal neuroimaging techniques including MRS, T_1_w/T_2_w ratio map, and rsfMRI, we examined how GSH levels, myelination, functional connectivity, and behavioral phenotypes showed specific association patterns in individuals with ASD. MRS data revealed that GSH levels in the left TPJ were significantly higher in the ASD group than in the TD group. We demonstrated that left TPJ GSH levels were positively associated with the left MFG T_1_w/T_2_w ratio, specifically in the ASD group. The correlations between whole‐brain functional connectivity patterns from the left MFG and left MFG T_1_w/T_2_w ratio were also significantly different between groups. Finally, we found ASD‐specific associations between fronto‐occipital functional connectivity and emotion recognition ability as assessed using a well‐validated cognitive task (RMET).

Contrary to our hypothesis, GSH levels in the left TPJ were higher in the ASD group than in the TD group, with a moderate effect size. As described in the Introduction, the left TPJ has not been investigated in previous MRS studies concerning ASD; thus, the difference that we observed may reflect region specificity. This finding seems to contradict those of postmortem brain studies that have revealed decreased GSH levels in the temporal cortex (Chauhan et al. 2012; Rose et al. 2012). One possibility may stem from differences in methodology itself. For example, glutathione levels in the postmortem brain could be affected by various premortem factors such as agonal state, medication status, or the cause of death (Calderón‐Guzmán et al. 2008; Harish et al. 2011). Previous postmortem brain studies may have partly reflected the effects of these factors. Therefore, the different results from the present study should be interpreted with caution. Another possible explanation for this discrepancy may be different participant characteristics. For example, these previous studies included data obtained from children, whereas this study included only adults. Metabolite concentrations assessed by MRS may follow different developmental trajectories between individuals with ASD and TD; for example, a recent MRS meta‐analysis showed that reduced *N*‐acetylaspartate levels in ASD were found in children but not in adults (Thomson et al. 2024). Thus, a significant group difference may be observed at certain developmental stages but not at others. Reversed patterns of group differences can also be observed in children and adults. Future studies are expected to confirm these age‐dependent differences using longitudinal MRS assessments.

In the ASD group, higher GSH levels in the left TPJ promoted more myelination in the left MFG. As the antioxidant effect of GSH may protect against oxidative damage to myelin (Bolaños et al. 1996; de la Asuncion et al. 1996; Sastre et al. 1996), the positive correlation between GSH levels and myelination, as indexed by the T_1_w/T_2_w ratio, seems reasonable. Studies have proposed oxidative stress, possibly reflected as reduced GSH levels or myelination, as a candidate biomarker for ASD (Chen et al. 2021; Frustaci et al. 2012; Khemakhem et al. 2017), but our findings suggest that such a negative impact is not necessarily present in the central nervous system of adults with ASD. Rather, elevated GSH levels and their positive association with increased myelination suggest a compensatory neural mechanism.

Interestingly, the result of the T_1_w/T_2_w map analysis suggests that altered GSH levels in one region (left TPJ) can affect myelination in a distal region (left MFG). An increase in local GSH levels could promote local myelination through reduced oxidative stress. This effect may spread to other brain regions via electrical signals transmitted through synaptic connections, given that myelination can be promoted by electrical neural activity (Wake et al. 2011). However, the underlying mechanisms by which region‐specific molecular alterations affect properties of structural or functional networks in spatially distant regions are highly complicated and have not been sufficiently investigated. Future studies should address these mechanisms.

MVPA revealed that the association between left MFG myelination and whole‐brain functional connectivity patterns from the left MFG differed significantly between the ASD and TD groups. Since increased myelin content may alter functional connectivity by speeding up the transmission of electrical signals, it is plausible that left MFG myelination and the functional connectivity patterns originating from the left MFG are interrelated in a specific manner in the ASD group. Notably, although MVPA does not rely on a priori assumptions about ROIs, the results from the T_1_w/T_2_w ratio map analysis and MVPA for rsfMRI data converged in the same region, the left MFG. Therefore, the left MFG may be a key region for a possible compensatory neural mechanism in the brains of individuals with ASD. Moreover, the post hoc seed‐to‐voxel analysis using the left MFG as a seed identified some regions included in the default mode network (DMN), such as the medial prefrontal cortex, precuneus, and bilateral inferior parietal lobule (Igelström and Graziano 2017; Menon 2023; Raichle 2015). The DMN overlaps with the mentalizing network, which is involved in inferring the mental states of others (Li et al. 2014; Mars et al. 2012). Accumulating evidence suggests that individuals with ASD often experience difficulty with mentalizing (Chung et al. 2014; Velikonja et al. 2019), which may be due to the atypical network properties of the DMN. Functional connectivity from the left MFG, where myelination increased along with increased GSH levels in the left TPJ, may have some facilitating effect on atypical DMN functioning in ASD; however, this is only speculation.

In the post hoc seed‐to‐voxel analysis, the left MFG seed and left fusiform gyrus seed generated spatially different patterns (Figures S2 and S3). As discussed above, the analysis using the left MFG seed showed that functional connectivity from the left MFG with some areas in the DMN, while, as for the analysis with the left fusiform gyrus seed, group differences in the correlation with the left MFG myelination were identified in other DMN nodes (i.e., a more rostral part of the medial prefrontal cortex, precuneus, bilateral TPJ). The fusiform gyrus, a crucial region for the processing of face information, has been implicated with characteristics of social information processing in individuals with ASD (Floris et al. 2025; Kuno‐Fujita et al. 2020). We therefore speculate that altered correlation patterns of the fusiform‐DMN connectivity with myelination may underlie social behavioral characteristics of individuals with ASD. Importantly, the analysis using the left fusiform seed also identified the left MFG, despite the data‐driven nature of this analysis. This finding further supports the significance of the left MFG in compensatory brain networks in individuals with ASD.

Increased functional connectivity between the left MFG and bilateral occipitoparietal cortex was associated with higher emotion recognition ability only in participants with ASD, and these occipitoparietal clusters were located adjacent to the TPJ. Previous studies have revealed increased activation near this occipitoparietal region when observing socio‐emotional pictures (Beyer et al. 2014) or inferring the intention of another person (Atique et al. 2011). These findings may support the view that increased functional connectivity from the left MFG to the occipitoparietal regions may have a compensatory effect on social functioning in individuals with ASD. Although we could not draw a clear conclusion on this point because we did not include task‐related fMRI assessments, further studies should investigate the possible associations between resting‐state neural characteristics (such as GSH levels, myelin maps, and rsFC) and task‐related activity in individuals with ASD.

Another possible interpretation of these interactions is from the ASD heterogeneity perspective; some researchers have suggested that ASD may consist of subgroups with different neurobiological or pathophysiological bases (Brunsdon and Happé 2014; Hong et al. 2018; Lombardo et al. 2019). For example, in a previous study, the researchers identified subgroups based on RMET performance (Lombardo et al. 2016). Herein, although a significant group difference was identified, some participants in the ASD group scored high, comparable to, or even higher than the average score of the TD group. Hypothetically, individuals with ASD who exhibit different behavioral characteristics may fall into two different subgroups—the high and low RMET subgroups—and only the low RMET subgroup would have lower functional connectivity between the left MFG and occipitoparietal cortex, while the high RMET subgroup would have a similar functional connectivity pattern to that of the TD participants. In such cases, participants with high and low RMET scores would show high and low functional connectivity, respectively, within the ASD group. Therefore, a positive pseudo‐correlation would be observed, which would reflect the existence of two subgroups in the ASD population. Future studies should investigate potentially conflicting explanations for these interactions.

This study has some limitations. First, the sample size was small, which limits the generalizability of the findings. Although we found increased GSH levels in the ASD group compared with the TD group, this result did not survive correction for multiple comparisons. Given the moderate effect size obtained in the MRS finding for the left TPJ (Cohen's *d* = 0.56) and the sample size (30 and 27 for each group), the achieved power was estimated as 0.545 (alpha = 0.05, two‐tailed). The post hoc power was low, indicating a limited probability of detecting a moderate effect. Future studies should estimate the required sample sizes and confirm these findings. Second, potential confounding factors could not be fully accounted for. For example, parental SES could not be matched between the groups. However, we believe that this difference had a minimal impact on our results, as the effects remained significant even after adjusting for it. Additionally, the high prevalence of comorbid psychiatric conditions, such as depression and anxiety, in individuals with ASD may be another source of confounding effects. However, we believe this effect was negligible because there was no difference in psychological distress between the groups (Table S1). Third, we did not control for medication effects.

In conclusion, we demonstrated ASD‐specific associations between GSH levels, myelination, functional connectivity, and cognitive phenotypes. Increased GSH levels in the left TPJ were positively associated with the left MFG T_1_w/T_2_w ratio, specifically in the ASD group. Moreover, the left MFG T_1_w/T_2_w ratio was differently correlated with whole‐brain functional connectivity patterns from the left MFG between the groups. Finally, we found ASD‐specific associations between functional connectivity and emotion recognition ability. These findings warrant further investigation of the compensatory neural mechanisms that support the social lives of individuals with ASD.

## Ethics Statement

This study was approved by the Ethics Committees of the University of Fukui (Protocol No. 20200112) and Hamamatsu University School of Medicine (Protocol No. 20‐258).

## Consent

Written informed consent was obtained from all participants.

## Conflicts of Interest

The authors declare no conflicts of interest.

## Supporting information


**Figure S1:** MRS data examples. (a) Example image of the ^1^H‐MRS regions of interest. (b) Sample spectrum obtained from an LCModel fitted result. White, the left temporoparietal junction; green, the right temporoparietal junction; red, the cerebellum; blue, the pregenual anterior cingulate cortex. ^1^H‐MRS, proton magnetic resonance spectroscopy.
**Figure S2:** Results of post hoc seed‐to‐voxel analysis using the left MFG seed. Functional connectivity between the purple regions and the left MFG seed was more positively correlated with the left MFG T_1_w/T_2_w ratio in the TD group than in the ASD group. The yellow region shows the opposite pattern of correlations. ASD, autism spectrum disorder; MFG, middle frontal gyrus; TD, typical development.
**Figure S3:** Results of post hoc seed‐to‐voxel analysis using the left fusiform seed. Functional connectivity between the purple regions and the left fusiform seed was more positively correlated with the left MFG T_1_w/T_2_w ratio in the TD group than in the ASD group. ASD, autism spectrum disorder; MFG, middle frontal gyrus; TD, typical development.
**Figure S4:** Comparison of plasma total glutathione levels between the ASD and TD groups. Plasma total glutathione levels in participants with TD (0.324 ± 0.166 μmol/L, *n* = 26) and ASD (0.419 ± 0.247 μmol/L, *n* = 29). Data are presented as the mean (± SD). Mann–Whitney *U* test. ASD, autism spectrum disorder; SD, standard deviation; TD, typical development.
**Table S1:** Participants' background information.
**Table S2:** Results of post hoc seed‐to‐voxel analysis using the left MFG and left fusiform gyrus as seed regions.
**Table S3:** Correlation between plasma total glutathione levels and brain GSH levels.
**Table S4:** Correlation between plasma total glutathione levels and ASD traits.

## Data Availability

The data that support the findings of this study are available from the corresponding author upon reasonable request.
