# Supplementary material for: Specific Association Patterns Between Brain Glutathione Levels, Myelination, and Functional Connectivity in Adults With Autism Spectrum Disorder
Source: Autism Res. 2025 Oct 23;18(12):2451–62. doi: 10.1002/aur.70134 (PMC12729515; doi:10.1002/aur.70134)
Supplement: Supplementary file 1 — Figure S1: MRS data examples. (a) Example image of the 1H‐MRS regions of interest. (b) Sample spectrum obtained from an LCModel fitted result. White, the left temporoparietal junction; green, the right temporoparietal junction; red, the cerebellum; blue, the pregenual anterior cingulate cortex. 1H‐MRS, proton magnetic resonance spectroscopy. Figure S2: Results of post hoc seed‐to‐voxel analysis using the left MFG seed. Functional connectivity between the purple regions and the left MFG seed was more positively correlated with the left MFG T1w/T2w ratio in the TD group than in the ASD group. The yellow region shows the opposite pattern of correlations. ASD, autism spectrum disorder; MFG, middle frontal gyrus; TD, typical development. Figure S3: Results of post hoc seed‐to‐voxel analysis using the left fusiform seed. Functional connectivity between the purple regions and the left fusiform seed was more positively correlated with the left MFG T1w/T2w ratio in the TD group than in the ASD group. ASD, autism spectrum disorder; MFG, middle frontal gyrus; TD, typical development. Figure S4: Comparison of plasma total glutathione levels between the ASD and TD groups. Plasma total glutathione levels in participants with TD (0.324 ± 0.166 μmol/L, n = 26) and ASD (0.419 ± 0.247 μmol/L, n = 29). Data are presented as the mean (± SD). Mann–Whitney U test. ASD, autism spectrum disorder; SD, standard deviation; TD, typical development. Table S1: Participants' background information. Table S2: Results of post hoc seed‐to‐voxel analysis using the left MFG and left fusiform gyrus as seed regions. Table S3: Correlation between plasma total glutathione levels and brain GSH levels. Table S4: Correlation between plasma total glutathione levels and ASD traits. [file AUR-18-2451-s001.docx]

**Supplementary Information for “Specific association patterns between brain glutathione levels, myelination, and functional connectivity in adults with autism spectrum disorder”**

**Supplementary Methods**

**Supplementary Results**

**Supplementary References**

**Supplementary Figure 1.** MRS data examples.

**Supplementary Figure 2.** Results of post-hoc seed-to-voxel analysis using the left MFG seed.

**Supplementary Figure 3.** Results of post-hoc seed-to-voxel analysis using the left fusiform seed.

**Supplementary Figure 4.** Comparison of plasma total glutathione levels between the ASD and TD groups.

**Supplementary Table 1.** Participants’ background information.

**Supplementary Table 2.** Results of post-hoc seed-to-voxel analysis using the left MFG and left fusiform gyrus as seed regions.

**Supplementary Table 3.** Correlation between plasma total glutathione levels and brain GSH levels.

**Supplementary Table 4.** Correlation between plasma total glutathione levels and ASD traits.

**Supplementary Methods**

*Supplementary Methods 1. Participants*

For additional background and clinical information, we collected data on height, body weight, smoking habits (yes or no), drinking habits (yes or no), psychological distress using the 12-item General Health Questionnaire (GHQ-12) (Goldberg, 1972; Goldberg & Williams, 1988; Toyabe et al., 2007), and severity of repetitive and restricted behaviors using the Repetitive Behavior Scale-Revised (RBS-R) (Bodfish, Symons, Parker, & Lewis, 2000; Inada et al., 2015). Participants were instructed to abstain from smoking for at least 72 hours before MRI data acquisition. We also asked about participants’ medication status, but this information was not included in the analysis because of the large individual differences in the type and number of medications and the difficulty in establishing a washout period.

*Supplementary Methods 2. MRI and MRS data acquisition*

T1-weighted images were collected with the following parameters: repetition time (TR) = 5.7 ms, echo time (TE) = 1.7 ms, inversion time = 400 ms, flip angle = 15°, slice thickness = 1 mm, voxel size = 1 × 1 × 1 mm^3^, matrix = 256 × 256, and 156 axial slices. For T2-weighted images, the following parameters were used: TR = 2000 ms, TE = 92.7 ms, flip angle = 90°, slice thickness = 1 mm, voxel size = 1 × 1 × 1 mm^3^, matrix = 512 × 512, and 180 sagittal slices.

To assess resting-state functional connectivity, T2*-weighted gradient-echo echo-planar images were acquired as rsfMRI data. The parameters were as follows: TR = 2500 ms, TE = 30 ms, flip angle = 80°, slice thickness = 3 mm with a 0.5 mm gap, voxel size = 3 × 3 × 3.5 mm^3^, matrix = 64 × 64, 42 transversal slices with an interleaved slice order, and 240 volumes. Participants were instructed to gaze at a black fixation cross on a light-gray background through a mirror mounted in front of their face during a 10-minute resting-state fMRI run.

To measure GSH levels in the brain, ^1^H-MRS was performed with the point-resolved spectroscopy sequence (PRESS; GE Medical Systems PROBE-P): voxel size = 2.5 cm × 2.5 cm × 2 cm, TE = 30 ms, TR = 1500 ms, spectral width = 5000, data points = 4096, number of excitations for water-suppressed data = 256, and number of excitations for unsuppressed water = 8. All four regions of interest (ROIs) were rectangular in shape, and the positions were manually set using axial, sagittal, and coronal slices displayed on the scanner console. The pgACC ROI was placed in the middle of the two hemispheres such that the back of the rectangle was tangential to the boundary between the corpus callosum and medial prefrontal cortex. For the TPJ ROI, we first identified the end of the Sylvian fissure of the brain of each individual, and then placed each ROI such that the end position and the temporal and parietal cortices were partially included in the rectangle. The cerebellum ROI was placed in the middle of the left and right hemispheres to fit within the cerebellum.

*Supplementary Methods 3. Cognitive phenotypic data acquisition and analysis*

*Supplementary Methods 3-1. Reading the Mind in the Eyes Test*

The Japanese version of the Reading the Mind in the Eyes Test (RMET) (Sato et al., 2016; Sato et al., 2017) was used to assess emotion recognition ability. In each trial, participants were presented with a photograph depicting the eye region of an Asian person along with four Japanese terms describing mental states (three foil terms and one target term; e.g., reflective, aghast, irritated, and impatient). Participants were required to orally select the target term that best described the emotion depicted. The test consisted of 36 trials, and responses were recorded by the experimenter (TI). Accuracy was used as a measure of emotion recognition ability.

*Supplementary Methods 3-2. Binocular rivalry task*

When two different visual stimuli are presented separately to the left and right eyes of the same person, one image is predominantly perceived for a certain period, while the other is suppressed. The dominant perception spontaneously alternates during viewing, with brief periods of mixed perception of these two images occurring between dominant phases. This phenomenon is known as binocular rivalry.

As experimental stimuli, we created 12 pairs of translucent, superimposed red and blue object images. In each trial, participants viewed one of these pairs through red-blue glasses, such that each eye received a different object image. While viewing a stimulus, participants were asked to indicate whether they perceived the red image, blue image, or their mixture by continuously pressing a 10-key keypad with the index (red), middle (blue), or ring (mixture) finger of their right hand.

Instructions and a practice trial were provided before the experiment. The red-blue image pair presented in the practice trial was not used in the experiment. The experiment comprised two sessions (six trials each) separated by a short break. During each session, a fixation cross surrounded by a circle was continuously displayed at the center of the monitor. A red-blue image pair appeared in the center of the monitor 2 seconds after participants pressed a specific key with their right little fingers. Each red-blue image pair was presented for 1 minute, during which participants gazed at the crosshair position and continuously reported their subjective perceptions by pressing a key, as described above. Stimulus presentation and response acquisition were controlled using Presentation software (Neurobehavioral Systems, Inc., Albany, CA, USA).

The perceptual switch rate (number of perceptual switches per trial) and proportion of perceptual suppression ([duration of red- or blue-dominant perception]/[duration of dominant perception + duration of mixed perception]) were used as indices of perceptual flexibility or rigidity (Robertson, Kravitz, Freyberg, Baron-Cohen, & Baker, 2013; Robertson, Ratai, & Kanwisher, 2016). To calculate the perceptual switch rate, transition events between different perceptual states (red-dominant, blue-dominant, or mixed) were detected as the termination of a continuous key-press and the initiation of another key-press. Using this event information, both perceptual switch rate and proportion of perceptual suppression were calculated.

*Supplementary Methods 3-3. Necker lattice task*

A Necker lattice composed of nine Necker cubes was used as the experimental stimulus (Kornmeier, Wörner, Riedel, & Tebartz van Elst, 2017). The Necker cube, a representative ambiguous figure, can be perceived in two different stereoscopic ways: the from-above and from-below perspectives. Participants viewed a Necker lattice at the center of the monitor for 3 minutes and continuously pressed a 10-key keypad with their right hand: the from-above perspective (index finger), from-below perspective (ring finger), or unclear perceptual interpretation (middle finger).

Participants received instructions before starting the experiment. After viewing the fixation cross at the center of the screen for 2 seconds, participants observed the Necker lattice and continuously reported their perception of the figure. The Presentation software (Neurobehavioral Systems, Inc.) was used to control the experiment.

As with the binocular rivalry task, perceptual transition events between the from-above perspective, from-below perspective, or unclear perceptual interpretation were identified based on the timing of keypress terminations and initiations. The perceptual switch rate (the number of perceptual switches per minute) was calculated and compared between the ASD and TD groups.

*Supplementary Methods 4. MRS data analysis*

After visual inspection of each spectrum, MRS data with Cramer–Rao lower bounds (%SD) > 20 were excluded from the analysis due to poor fitting quality. Full-width at half-maximum (FWHM) and signal-to-noise ratio (S/N) were obtained as additional quality measures. Using Gannet 3.1 (Edden, Puts, Harris, Barker, & Evans, 2014), the fractional volumes of gray matter (fGM), white matter (fWM), and cerebrospinal fluid (fCSF) within each ROI were estimated. The tissue composition in each ROI was corrected using the following equation: ${(GSH level)}/\left( 1-fCSF \right)$ (Aoki et al., 2012; Lutkenhoff et al., 2010). For each region, we conducted a two-sample *t*-test to examine group differences in GSH levels.

*Supplementary Methods 5. Myelin map analysis*

The analytical pipeline of the MRTool comprises the following steps (Ganzetti Wenderoth, & Mantini, 2014, 2015): First, using a rigid-body transformation, an individual T2-weighted image was co-registered with the T1-weighted image of the same participant. Second, bias correction was applied to the T1- and T2-weighted images. Third, both images were subjected to intensity standardization. Finally, the ratio between the calibrated T1- and T2-weighted images (T1w/T2w ratio) was calculated to obtain the individual myelin map. Finally, these individual myelin maps were spatially transformed into the Montreal Neurological Institute (MNI) space.

*Supplementary Methods 6. Resting-state fMRI data preprocessing*

After discarding the first four volumes to avoid T1 equilibrium effects, the remaining functional data (236 volumes) were realigned, corrected for slice timing, spatially normalized to the MNI space, and smoothed using a 6 mm full-width half-maximum kernel. Using the denoising pipeline implemented in the CONN toolbox, temporal band-pass filtering (0.008–0.09 Hz) was performed. To remove physiological and motion-related artifacts, we regressed out signals from white matter and cerebrospinal fluid along with motion outliers (with a volume-to-volume threshold of 0.9 mm and a global mean intensity threshold of 5 SDs) and Friston 24 head-motion parameters (Friston Williams, Howard, Frackowiak, & Turner, 1996). No significant differences were observed in mean frame-wise motion (*t* = 0.071, *p* = 0.94) and number of ineligible volumes (*t* = 1.05, *p* = 0.30) between the groups.

*Supplementary Methods 7. Multivariate pattern analysis: associations between T1w/T2w ratio and resting-state functional connectivity*

As mentioned in the main text, MVPA is a method for characterizing whole-brain functional connectivity patterns from each voxel using dimensionality reduction via singular value decomposition. In the present study, the first 10 components obtained by singular value decomposition were retained for group-level analysis to maintain an approximate 1:5 ratio for the number of components and sample size. We considered the number of retained components appropriate because these components explained 87.3% and 78.2% of the variance in gray matter and white matter, respectively.

A multivariate analysis of covariance was performed to examine the interaction effect between myelination (indexed as the T1w/T2w ratio) and the groups (with or without ASD) on whole-brain functional connectivity patterns. To create independent variables, we extracted T1w/T2w ratios from regions showing significant group × GSH interactions in the myelin map analysis. For this purpose, we used the *get_totals* MATLAB function (<http://www0.cs.ucl.ac.uk/staff/g.ridgway/vbm/get_totals.m>). Then, T1w/T2w values extracted from individual myelin maps, a proxy for regional myelination, were used to generate an independent variable in the MVPA. Along with the categorical group variables, the interaction terms between the T1w/T2w ratio and groups were included in the model to explore regions in which whole-brain functional connectivity patterns were differently associated with the T1w/T2w ratio between the groups. The statistical map, masked inclusively by the gray matter structure, was initially thresholded at voxel-level *p* < 0.001 (uncorrected) and then at cluster-level *p* < 0.05 (false-discovery rate corrected for multiple comparisons).

After identifying significant clusters using MVPA, we performed a post-hoc seed-to-voxel analysis using these clusters as seed regions to further illustrate the interaction between T1w/T2w ratio and ASD diagnosis on the functional connectivity between these seed regions and the rest of the brain. For the post-hoc analysis, a more conservative cluster-level thresholding was used (voxel-level threshold of *p* < 0.001 [uncorrected] and cluster-level threshold of *p* < 0.05 [family-wise error–corrected], two-tailed).

*Supplementary Methods 8. Plasma sample analysis*

Blood samples were collected from all the participants using vacuum blood collection tubes containing EDTA-2K. The samples were immediately centrifuged at 3500 rpm for 10 minutes using a refrigerated centrifuge, and the supernatant was collected as plasma samples. To measure total glutathione (GSSG/GSH), the obtained plasma samples were deproteinized with 5% metaphosphoric acid, left on ice for 15 minutes, and then centrifuged at 12,000 rpm for 10 minutes at 4 °C. The supernatant was aliquoted into microtubes of 200 μL each and stored at -80°C until further use.

Total glutathione levels were quantified using the OxiSelect™ Total Glutathione (GSSG/GSH) Assay Kit (Cell Biolabs, USA) per manufacturer instructions and measured with the SpectraMax iD5 spectrophotometric reader (Molecular Devices, San Jose, CA) using a colorimetric assay. All plasma samples were quantified in triplicate, and the mean values of the three measurements were used for analysis. Analytical values below the limit of detection were excluded from the analysis.

**Supplementary Results**

*Supplementary Results 1. Final sample for the statistical analysis*

Of the 32 participants recruited for the ASD group, the data of two were excluded from the analysis because their ADOS scores were below the cutoff. In the TD group, data acquisition was discontinued for two participants because of poor physical conditions; consequently, their data were excluded from the analysis. Further, the data of 16 participants in the TD group were excluded from the analysis because their SRS-2 scores exceeded the cutoff. The data of 27 TD participants were included in the final analysis. Supplementary Table 1 shows additional background information.

*Supplementary Results 2. Results of cognitive phenotypic data analysis*

Owing to difficulties in maintaining attention during the task, four participants in the ASD group did not complete the cognitive tasks. The data of the remaining participants (26 ASD and 27 TD) were included in the subsequent analyses.

*Supplementary Results 2-1. Reading the Mind in the Eyes Test*

The means±SDs of the RMET scores were 56.9±12.9% and 72.5±7.4% for the ASD and TD groups, respectively. A two-sample *t*-test revealed significant group differences (*t* = 5.43, *df* = 51, *p* < 0.001, Cohen’s *d* = 1.48).

*Supplementary Results 2-2. Binocular rivalry task*

The means±SDs of the perceptual switch rates and the proportions of perceptual suppression were as follows: perceptual switch rate, 5.49±3.23 for the ASD group, 5.85±2.24 for the TD group; proportion of perceptual suppression, 0.72±0.28 for the ASD group, 0.73±0.26 for the TD group. No significant group differences were observed for either measure (perceptual switch rate, *t* = 0.48, *df* = 51, *p* = 0.63, Cohen’s *d* = 0.13; proportion of perceptual suppression, *t* = 0.07, *df* = 51, *p* = 0.95, Cohen’s *d* = 0.04).

*Supplementary Results 2-3. Necker lattice task*

The means±SDs of the perceptual switch rate per minute in the Necker lattice task were 4.19±3.36 for the ASD group and 5.56±3.08 for the TD group. No significant group differences were observed (*t* = 1.54, *df* = 51, *p* = 0.13, Cohen’s *d* = 0.43).

*Supplementary Results 3. Sensitivity analysis results*

*Supplementary Text 3-1. Sensitivity analysis for MRS data*

We conducted a logistic regression analysis using the binary group variable (ASD = 1, TD = 0) as the dependent variable and left TPJ GSH levels as an independent variable. Parental SES, which differed significantly between the groups, was included as a covariate. We used STATA MP 17.0 (StataCorp, College Station, TX, USA) to perform this analysis. This analysis confirmed that higher levels of left TPJ GSH were significantly associated with an increased likelihood of being in the ASD group (odds ratio [OR] = 4.90, 95% confidence interval [CI] = 1.07 to 22.4, *p* = 0.04), even after adjusting for parental SES (OR = 0.23, 95% CI = 0.07 to 0.75, *p* = 0.02). The likelihood ratio test indicated a significant model fit (log likelihood = -29.7, *χ^2^* = 12.6, *p* = 0.002), with a pseudo R^2^ of 0.18.

*Supplementary Results 3-2. Sensitivity analysis for T1w/T2w ratio map*

For the sensitivity analysis, we performed a general linear model (GLM) analysis with four regressors: two categorical variables for either the ASD or the TD group, parental SES, and the interaction term between the group and left TPJ GSH levels. This additional GLM analysis confirmed the presence of the same left MFG region (peak coordinate = [-26, 19, 42], cluster size = 121). Statistical thresholds were *p* < 0.001 at the peak level (uncorrected for multiple comparisons, one-tailed) and *p* < 0.05 (false-discovery rate–corrected, one-tailed) at the cluster level.

*Supplementary Results 3-3. Sensitivity analysis for MVPA*

After including parental SES as a covariate, multivariate analysis of covariance revealed that the left MFG (peak coordinate = [-32, 38, 44], cluster size = 30) and the left fusiform gyrus (peak coordinate = [-28, -34, -22], cluster size = 43) were involved in the interaction between the left MFG T1w/T2w ratio and the group (*p* < 0.001 uncorrected at the peak level and *p* < 0.05 false-discovery rate–corrected at the cluster level). The left orbitofrontal cluster was not identified in this sensitivity analysis. Several other regions also showed significant interactions: the right superior frontal gyrus (peak coordinate = [18, 6, 66], cluster size = 48), subcallosal cortex (peak coordinate = [0, 14, -16], cluster size = 42), and right fusiform gyrus (peak coordinate = [34, -18, -38], cluster size = 24).

*Supplementary Results 3-4. Sensitivity analysis for associations between rsFC and cognitive phenotypes*

The sensitivity analysis confirmed that the right (peak coordinate = [46, -68, 28], cluster size 41) and left (peak coordinate = [-38, -70, 20], cluster size = 30) occipitoparietal junctions showed a significant interaction between emotion recognition ability (i.e., RMET accuracy) and the groups only when a liberal threshold was used (*p* < 0.001, uncorrected for multiple comparisons). No significant activation was observed after false-discovery rate correction for multiple comparisons. This may be due to the reduced sample size in the sensitivity analysis. However, because almost the same clusters were detected in the bilateral occipitoparietal junction as in the main analysis, we believe that the results are reliable.

*Supplementary Results 4. Results of plasma sample analysis*

No significant differences were observed in plasma total glutathione levels between the ASD and TD groups (Supplementary Figure 4). Plasma total glutathione levels were not associated with brain GSH levels as measured by ^1^H-MRS (Supplementary Table 3). No significant correlation was found between plasma total glutathione levels and ASD traits as assessed by questionnaires (Supplementary Table 4).

**Supplementary References**

Aoki, Y., Abe, O., Yahata, N., Kuwabara, H., Natsubori, T., Iwashiro, N., . . . Yamasue, H. (2012). Absence of age-related prefrontal NAA change in adults with autism spectrum disorders. *Translational Psychiatry*, *2*(10), e178. doi:[10.1038/tp.2012.108](https://doi.org/10.1038/tp.2012.108).

Bodfish, J. W., Symons, F. J., Parker, D. E., & Lewis, M. H. (2000). Varieties of repetitive behavior in autism: comparisons to mental retardation. *Journal of Autism and Developmental Disorders*, *30*(3), 237–243. doi:[10.1023/a:1005596502855](https://doi.org/10.1023/a:1005596502855).

Edden, R. A., Puts, N. A., Harris, A. D., Barker, P. B., & Evans, C. J. (2014). Gannet: A batch-processing tool for the quantitative analysis of gamma-aminobutyric acid-edited MR spectroscopy spectra. *Journal of Magnetic Resonance Imaging*, *40*(6), 1445–1452. doi:[10.1002/jmri.24478](https://doi.org/10.1002/jmri.24478).

Friston, K. J., Williams, S., Howard, R., Frackowiak, R. S., & Turner, R. (1996). Movement-related effects in fMRI time-series. *Magnetic Resonance in Medicine*, *35*(3), 346–355. doi:[10.1002/mrm.1910350312](https://doi.org/10.1002/mrm.1910350312).

Ganzetti, M., Wenderoth, N., & Mantini, D. (2014). Whole brain myelin mapping using T1- and T2-weighted MR imaging data. *Frontiers in Human Neuroscience*, *8*, 671. doi:[10.3389/fnhum.2014.00671](https://doi.org/10.3389/fnhum.2014.00671).

Ganzetti, M., Wenderoth, N., & Mantini, D. (2015). Mapping pathological changes in brain structure by combining T1- and T2-weighted MR imaging data. *Neuroradiology*, *57*(9), 917–928. doi:[10.1007/s00234-015-1550-4](https://doi.org/10.1007/s00234-015-1550-4).

Goldberg, D. P. (1972). *The detection of psychiatric illness by questionnaire. Maudsley Monograph no. 21*. London: Oxford University Press.

Goldberg, D. P., & Williams, P. A. (1988). *A User’s guide to the general health questionnaire*. Basingstoke: NFER-Nelson.

Inada, N., Ito, H., Yasunaga, K., Kuroda, M., Iwanaga, R., Hagiwara, T., . . . Tsujii, M. (2015). Psychometric properties of the Repetitive Behavior Scale-Revised for individuals with autism spectrum disorder in Japan. *Research in Autism Spectrum Disorders*, *15–16*, 60–68. doi:[10.1016/j.rasd.2015.01.002](https://doi.org/10.1016/j.rasd.2015.01.002).

Kornmeier, J., Wörner, R., Riedel, A., & Tebartz van Elst, L. (2017). A different view on the Necker cube-Differences in multistable perception dynamics between Asperger and non-Asperger observers. *PLOS One*, *12*(12), e0189197. doi:[10.1371/journal.pone.0189197](https://doi.org/10.1371/journal.pone.0189197).

Lutkenhoff, E. S., van Erp, T. G., Thomas, M. A., Therman, S., Manninen, M., Huttunen, M. O., . . . Cannon, T. D. (2010). Proton MRS in twin pairs discordant for schizophrenia. *Molecular Psychiatry*, *15*(3), 308–318. doi:[10.1038/mp.2008.87](https://doi.org/10.1038/mp.2008.87).

Robertson, C. E., Kravitz, D. J., Freyberg, J., Baron-Cohen, S., & Baker, C. I. (2013). Slower rate of binocular rivalry in autism. *Journal of Neuroscience*, *33*(43), 16983-16991. https://doi.org/10.1523/JNEUROSCI.0448-13.2013

Robertson, C. E., Ratai, E. M., & Kanwisher, N. (2016). Reduced GABAergic action in the autistic brain. *Current Biology*, *26*(1), 80–85. doi:[10.1016/j.cub.2015.11.019](https://doi.org/10.1016/j.cub.2015.11.019).

Sato, W., Kochiyama, T., Uono, S., Sawada, R., Kubota, Y., Yoshimura, S., & Toichi, M. (2016). Structural neural substrates of reading the mind in the eyes. *Frontiers in Human Neuroscience*, *10*, 151. doi:[10.3389/fnhum.2016.00151](https://doi.org/10.3389/fnhum.2016.00151).

Sato, W., Uono, S., Kochiyama, T., Yoshimura, S., Sawada, R., Kubota, Y., . . . Toichi, M. (2017). Structural correlates of reading the mind in the eyes in autism spectrum disorder. *Frontiers in Human Neuroscience*, *11*, 361. doi:[10.3389/fnhum.2017.00361](https://doi.org/10.3389/fnhum.2017.00361).

Toyabe, S., Shioiri, T., Kobayashi, K., Kuwabara, H., Koizumi, M., Endo, T., . . . Akazawa, K. (2007). Factor structure of the General Health Questionnaire (GHQ-12) in subjects who had suffered from the 2004 Niigata-Chuetsu Earthquake in Japan: a community-based study. *BMC Public Health*, *7*, 175. doi:[10.1186/1471-2458-7-175](https://doi.org/10.1186/1471-2458-7-175).


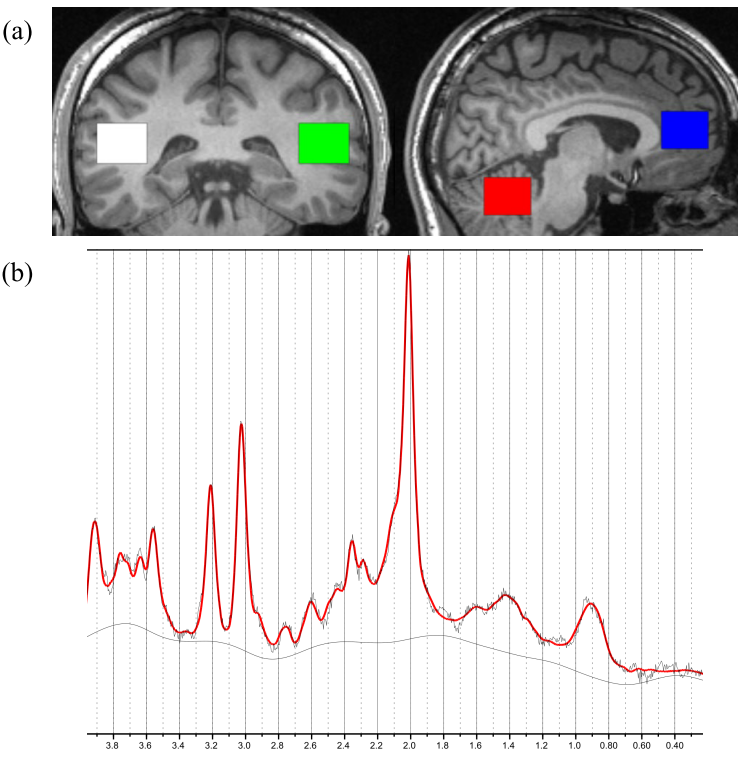


**Supplementary Figure 1.** MRS data examples. (a) Example image of the ^1^H-MRS regions of interest. (b) Sample spectrum obtained from an LCModel fitted result. White, the left temporoparietal junction; green, the right temporoparietal junction; red, the cerebellum; blue, the pregenual anterior cingulate cortex. ^1^H-MRS, proton magnetic resonance spectroscopy.


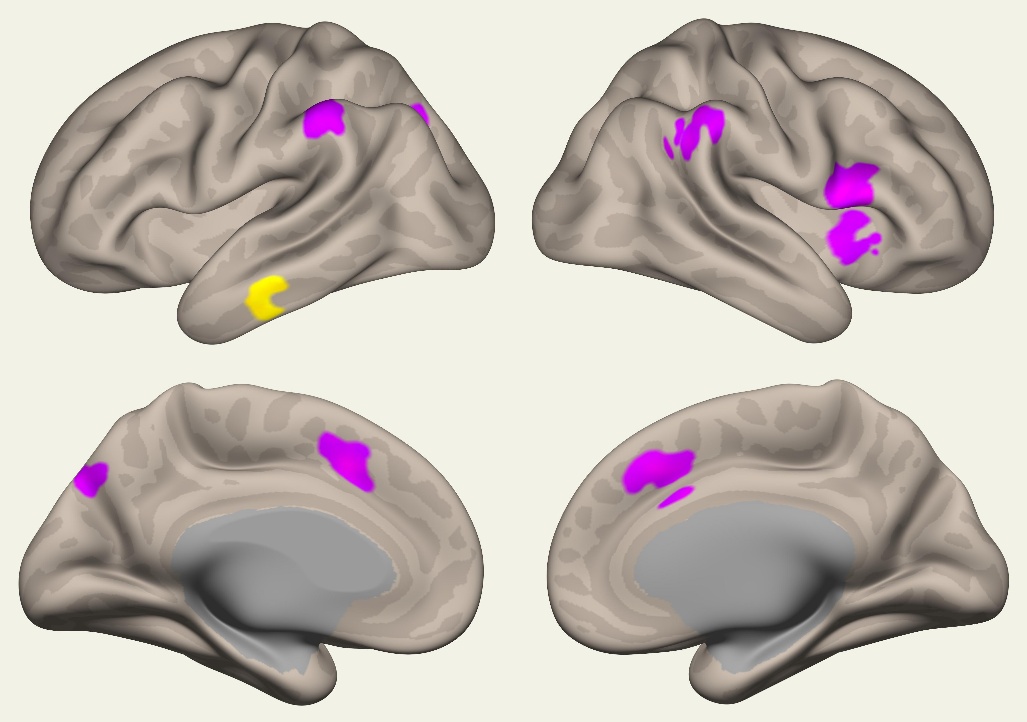


**Supplementary Figure 2.** Results of post-hoc seed-to-voxel analysis using the left MFG seed. Functional connectivity between the purple regions and the left MFG seed was more positively correlated with the left MFG T1w/T2w ratio in the TD group than in the ASD group. The yellow region shows the opposite pattern of correlations. MFG, middle frontal gyrus; ASD, autism spectrum disorder; TD, typical development.


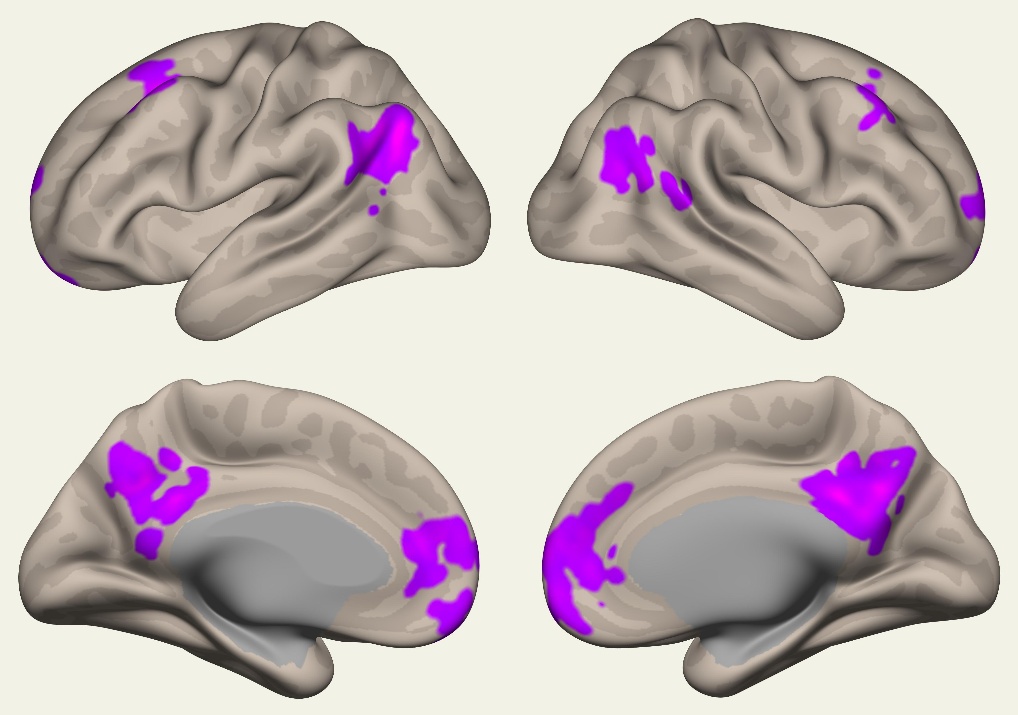


**Supplementary Figure 3.** Results of post-hoc seed-to-voxel analysis using the left fusiform seed. Functional connectivity between the purple regions and the left fusiform seed was more positively correlated with the left MFG T1w/T2w ratio in the TD group than in the ASD group. MFG, middle frontal gyrus; ASD, autism spectrum disorder; TD, typical development.


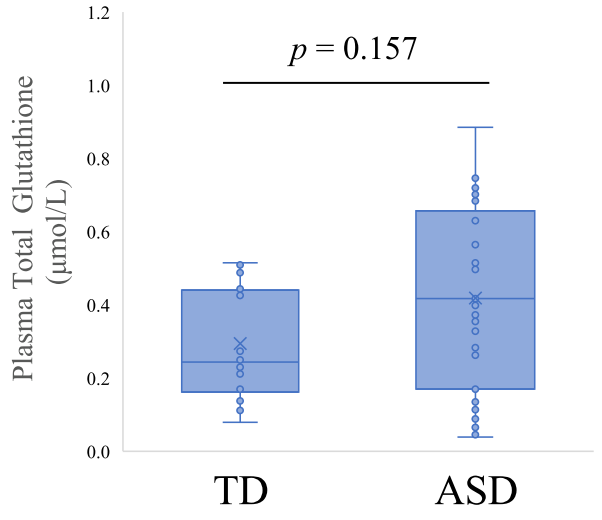


**Supplementary Figure 4.** Comparison of plasma total glutathione levels between the ASD and TD groups. Plasma total glutathione levels in participants with TD (0.324 ± 0.166 μmol/L, n=26) and ASD (0.419 ± 0.247 μmol/L, n=29). Data are presented as the mean (± SD). Mann–Whitney U test. ASD, autism spectrum disorder; TD, typical development, SD, standard deviation.

**Supplementary Table 1.** Participants’ background information.

|  | ASD (N =30) | TD (N =27) | Statistics |
| --- | --- | --- | --- |
| Characteristics | Mean (SD) | Mean (SD) |  |
| Height | 167.6 (7.9) | 168.1 (8.0) | *t* = 0.25, *p* = 0.80 |
| Body weight | 62.9 (11.6) | 65.1 (12.6) | *t* = 0.67, *p* = 0.51 |
| Smoking habit (# of yes) | 0 | 4 | *χ^2^* = 4.78, *p* = 0.02 |
| Drinking habit (# of yes) | 13 | 19 | *χ^2^* = 4.29, *p* = 0.11 |
| GHQ12 | 3.0 (3.0) | 2.8 (2.6) | *t* = 0.34, *p* = 0.73 |
| RBS-R (total score) | 10.6 (13.1) | 1.6 (3.7) | *t* = 3.56. *p* = 0.001 |

ASD, autism spectrum disorder; TD, typical development; SD, standard deviation; GHQ12, 12-item General Health Questionnaire; RBS-R, Repetitive Behavior Scale-Revised.

**Supplementary Table 2**. Results of post-hoc seed-to-voxel analysis using the left MFG and left fusiform gyrus as seed regions.

|  | MNI coordinates | | |  |  |
| --- | --- | --- | --- | --- | --- |
| Region label | *x* | *y* | *z* | *t*-value | *k* |
| L MFG seed |  |  |  |  |  |
| R putamen | 26 | 16 | 2 | -6.44 | 320 |
| L/R ACC/DMPFC | -8 | 20 | 42 | -6.10 | 458 |
| R IFG, pars opercularis | 52 | 16 | 14 | -5.85 | 266 |
| R supramarginal gyrus | 58 | -44 | 36 | -5.15 | 194 |
| L middle temporal gyrus | -68 | -16 | -22 | 5.14 | 104 |
| L supramarginal gyrus | -64 | -42 | 42 | -4.86 | 293 |
| L lateral occipital cortex/precuneus | -20 | -74 | 38 | -4.79 | 104 |
| L fusiform gyrus seed |  |  |  |  |  |
| L/R anterior MPFC | 0 | 56 | 12 | -7.29 | 1385 |
| L MFG | -20 | 18 | 44 | -7.12 | 495 |
| L/R cerebellum | -6 | -62 | -50 | -6.58 | 386 |
| L occipitoparietal junction | -46 | -74 | 42 | -6.53 | 818 |
| L/R precuneus/PCC | 0 | -48 | 36 | -6.42 | 1622 |
| R MFG | 40 | 26 | 48 | -6.15 | 283 |
| R occipitoparietal junction | 48 | -54 | 20 | -5.82 | 497 |
| L cerebellum | -8 | -84 | -26 | -5.71 | 202 |

L, left; R, right; MFG, middle frontal gyrus, ACC, anterior cingulate cortex; DMPFC, dorsomedial prefrontal cortex; IFG, inferior frontal gyrus; MPFC, medial prefrontal cortex; PCC, posterior cingulate cortex.

Note: Negative *t*-values indicate stronger correlations between left MFG myelination and functional connectivity with the seed region in the TD group than in the ASD group; positive *t*-values indicate the opposite correlation pattern.

**Supplementary Table 3**. Correlation between plasma total glutathione levels and brain GSH levels.

|  | ASD | TD | All participants |
| --- | --- | --- | --- |
| Region of interest |  |  |  |
| L TPJ | *r* = 0.18  (-0.20 to 0.51)  *p* = 0.36 | *r* = 0.05  (-0.34 to 0.42)  *p* = 0.81 | *r* = 0.19  (-0.07 to 0.43)  *p* = 0.16 |
| R TPJ | *r* = 0.15  (-0.22 to 0.49)  *p* = 0.43 | *r* = 0.28  (-0.12 to 0.60)  *p* = 0.17 | *r* = 0.24  (-0.03 to 0.47)  *p* = 0.08 |
| Cerebellum | *r* = -0.04  (-0.39 to 0.32)  *p* = 0.84 | *r* = -0.26  (-0.58 to 0.13)  *p* = 0.20 | *r* = -0.11  (-0.36 to 0.16)  *p* = 0.43 |

ASD, autism spectrum disorder; TD, typical development; R, right; L, left, TPJ, temporoparietal junction.

Note: Numbers in parentheses are 95% confidence intervals.

**Supplementary Table 4**. Correlation between plasma total glutathione levels and ASD traits.

|  | Plasma total glutathione  (*N* = 55) | | |
| --- | --- | --- | --- |
| ASD traits | *r* | 95% CI | *p* |
| SRS-2 | 0.14 | −0.14 to 0.40 | 0.31 |
| Total score | 0.16 | −0.11 to 0.42 | 0.23 |
| Awareness | 0.22 | −0.06 to 0.46 | 0.11 |
| Cognition | 0.12 | −0.16 to 0.38 | 0.38 |
| Communication | 0.09 | −0.18 to 0.36 | 0.49 |
| Motivation | 0.11 | −0.17 to 0.37 | 0.44 |
| Restricted interests and repetitive behavior | 0.14 | −0.14 to 0.40 | 0.31 |
|  |  |  |  |
| RBS-R |  |  |  |
| Total score | 0.23 | −0.04 to 0.47 | 0.09 |
| Stereotyped behavior subscale score | 0.08 | −0.20 to 0.34 | 0.57 |
| Self-injurious behavior subscale score | 0.15 | −0.13 to 0.40 | 0.29 |
| Compulsive subscale score | 0.11 | −0.17 to 0.37 | 0.42 |
| Ritualistic/sameness subscale score | 0.15 | −0.13 to 0.40 | 0.28 |
| Sameness behavior subscale score | 0.26 | −0.01 to 0.50 | 0.06 |
| Restricted behavior subscale score | 0.24 | −0.03 to 0.48 | 0.08 |

CI, confidence interval; SRS-2, Social Responsiveness Scale, Second Edition; RBS-R, Repetitive Behavior Scale-Revised.
